# Salivary glands as the primary dose-limiting organ: an integrated dosimetry, efficacy, and toxicity study of ^225^Ac-PSMA-617 in prostate cancer xenografts

**DOI:** 10.3389/fphar.2026.1807913

**Published:** 2026-04-10

**Authors:** Chenchen Cai, Peishang Li, Zhiqian Wang, Yiling Chen, Chunmiao Qu, Chunlei Jin, Xiangsheng Kong

**Affiliations:** 1 School of Pharmacy and Laboratory of Drug Discovery from Natural Resources and Industrialization, Macau University of Science and Technology, Macau, China; 2 Mednovo Group Co., Ltd., Suzhou, Jiangsu, China

**Keywords:** ^225^Ac-PSMA-617, dose-response, dosimetry, salivary gland toxicity, targeted alpha therapy, therapeutic index

## Abstract

**Background:**

Targeted alpha therapy with ^225^Ac-PSMA-617 is a promising strategy for metastatic prostate cancer. However, physiological uptake in dose-limiting organs, particularly the salivary glands and kidneys, necessitates precise dosimetry to define the therapeutic window. Therefore, the objective of this study was to perform integrated dosimetry, quantify the therapeutic index (TI), and establish dose-response relationships for the tumor control and organ toxicity of ^225^Ac-PSMA-617 in a PSMA-positive xenograft model.

**Methods:**

In male nude mice bearing C4-2 xenografts, a dosimetry group (n = 32) received 5 kBq ^225^Ac-PSMA-617 for multi-time-point biodistribution. Time-activity curves were fitted with bi-exponential models, and absorbed doses were calculated using the MIRD formalism. A therapy group (n = 12/arm) received a single 40 kBq dose of the therapy or vehicle. Efficacy was assessed by tumor growth inhibition (TGI) and time-to-progression (TTP) of 500 mm^3^. Toxicity was evaluated via renal function, histopathology (kidney: Day 56; salivary glands: Day 28), and pilocarpine-stimulated salivary secretion. The therapeutic index (TI_min) and Spearman correlations between absorbed dose and biological endpoints were calculated.

**Results:**

The radiopharmaceutical demonstrated high purity and stability. Dosimetry revealed favorable pharmacokinetics, with the highest absorbed dose coefficient for the tumor (588 Gy/GBq). The mean absorbed dose to the parotid gland (0.56 Gy at 5 kBq) exceeded that to the kidneys (0.29 Gy). The TI_min was >1 for all animals. The 40 kBq dose induced significant tumor growth inhibition (TGI: 75.2%, P < 0.001) and delayed progression (P < 0.001). While renal function remained normal, histology revealed mild tubular injury. Salivary gland function, however, showed a profound and sustained decrease (>60%, P < 0.001). Strong correlations were found between tumor dose and TGI (ρ = 0.720), kidney dose and histology score (ρ = 0.643), and salivary gland dose and functional impairment (ρ = 0.776) (all P < 0.05).

**Conclusion:**

^225^Ac-PSMA-617 provides a significant therapeutic window (TI > 1) with potent efficacy. The salivary glands, not the kidneys, are identified as the primary dose-limiting organ based on severe functional toxicity strongly correlated with absorbed dose. These quantitative dose-response correlations establish a foundation for clinical dose optimization.

## 1 Introduction

Prostate cancer remains a leading cause of cancer-related mortality in men, with metastatic castration-resistant prostate cancer (mCRPC) representing a particularly lethal and treatment-refractory stage (Posdzich et al., 2023; Swami et al., 2020). The prostate-specific membrane antigen (PSMA) is a well-validated therapeutic target, as it is highly overexpressed on the membrane of most prostate cancer cells (Maes et al., 2024; Corpetti et al., 2024). This expression has been successfully exploited with radioligand therapies (RLT), most notably with the beta-emitter ^177^Lu-PSMA-617, which has demonstrated significant clinical benefit. However, challenges including heterogeneous responses, intrinsic or acquired resistance, and disease relapse highlight the need for more potent treatment modalities (Sartor et al., 2021).

Targeted alpha therapy (TAT) with ^225^Ac-PSMA-617 represents a paradigm-shifting advance, leveraging the high linear energy transfer (LET) and short range of alpha particles to induce potent, localized cytotoxicity in PSMA-positive cells (Kairemo et al., 2024; Juzeniene et al., 2021). Early clinical results are promising, but this enhanced efficacy is coupled with a distinct toxicity profile (Ma et al., 2022). Physiological PSMA expression in proximal renal tubules and salivary glands results in unavoidable radionuclide accumulation, making these organs primary sites of dose-limiting toxicity (Heynickx et al., 2023). Xerostomia, resulting from salivary gland irradiation, is a frequent and clinically impactful adverse event that can compromise patient quality of life and treatment adherence (Muniz et al., 2024).

Defining the therapeutic window for ^225^Ac-PSMA-617 requires precise dosimetry to balance tumor control and organ-at-risk toxicity. However, dosimetry for alpha-emitters is inherently complex (Tronchin et al., 2022). Challenges include the multi-step decay chain of ^225^Ac, atomic recoil effects that can alter the local distribution of daughter nuclides, and the inability to directly image ^225^Ac with conventional clinical gamma cameras (Tronchin et al., 2022; Kruijff et al., 2019). Consequently, organ risk cannot be accurately extrapolated from administered activity alone, and microdosimetric effects may drive toxicity even when macroscopic mean absorbed doses appear modest (Sgouros et al., 2010).

Despite this clinical imperative, a critical translational gap persists in the preclinical literature. While studies have reported either the promising efficacy or the concerning toxicity of ^225^Ac-PSMA-617, a comprehensive framework that integrates detailed dosimetry, including full time-activity curves for tumor and all major organs at risk, with longitudinal functional and histopathological toxicity readouts under a unified protocol is lacking (Sathekge et al., 2021). Specifically, the concurrent quantification of absorbed doses, calculation of a robust therapeutic index, correlation of these physical metrics with biological endpoints, and rigorous assessment of dosimetric uncertainty are required to bridge the gap between preclinical models and clinical trial design (O’Donoghue et al., 2022).

Therefore, we conducted a preclinical study designed to address this gap. Using a PSMA-positive C4-2 xenograft model in nude mice, we synthesized quality-controlled ^225^Ac-PSMA-617 and performed detailed biodistribution studies. Our primary objectives were to: (i) quantify the absorbed doses to tumors, kidneys, and salivary glands and calculate the therapeutic index; (ii) evaluate the corresponding antitumor efficacy and organ-specific toxicities (renal and salivary); and (iii) establish correlations between absorbed dose and biological endpoints to identify the primary dose-limiting organ and provide a quantitative basis for defining the therapeutic window.

## 2 Materials and methods

### 2.1 Study design overview

This study was designed to evaluate the absorbed dose, therapeutic index, efficacy, and organ-specific toxicity of ^225^Ac-PSMA-617 in a PSMA-positive prostate cancer xenograft model. It comprised two main arms: a dosimetry group (n = 32) for biodistribution and dose calculation, and a therapy group (n = 24, split into treatment and vehicle control arms) for efficacy and toxicity assessment. Detailed information on all materials, reagents, cell lines, animals, and equipment, including suppliers and catalog numbers, is provided in Supplementary Table S1.

### 2.2 Radiopharmaceutical preparation and characterization

#### 2.2.1 ^225^Ac source and handling

^225^Ac was obtained as ^225^Ac(NO_3_)_3_ in 0.04 M HCl from a national atomic energy agency-licensed supplier. The activity concentration was verified using a traceable dose calibrator. Radionuclidic purity was confirmed by high-purity germanium gamma spectrometry, ensuring the characteristic peaks of ^225^Ac daughters ^221^Fr (∼218 keV) and ^213^Bi (∼440 keV) were present and that the ^227^Ac impurity was ≤0.1%. All work with ^225^Ac was conducted in accordance with strict alpha-radiation safety protocols, using disposable shielded equipment and continuous contamination monitoring.

#### 2.2.2 Synthesis of ^225^Ac-PSMA-617

The chelator-conjugated peptide PSMA-617 (DOTA-PSMA-617) was dissolved in sterile 0.1 M acetate buffer (pH 5.5). The ^225^Ac solution was added to achieve a ligand-to-metal molar ratio of 5:1 in a total reaction volume of 50–100 µL. The mixture was incubated at 95 °C for 45 min. To sequester unchelated ^225^Ac, diethylenetriaminepentaacetic acid (DTPA) was added to a final concentration of 25 µM. The final product was sterile-filtered (0.22 µm) and diluted to injection volume with 10% (v/v) ethanol in saline.

#### 2.2.3 Quality control and stability

Radiochemical purity (RCP) was assessed by instant thin-layer chromatography (iTLC-SG) and reverse-phase high-performance liquid chromatography (HPLC). For iTLC-SG, 0.1 M citrate buffer (pH 5.0) was used as the mobile phase; ^225^Ac-PSMA-617 remained at the origin (Rf ∼0.0–0.2) while ^225^Ac-DTPA migrated with the solvent front. HPLC was performed on a C18 column with a gradient of 0.1% trifluoroacetic acid in water and acetonitrile, with simultaneous UV (220 nm) and in-line radioactivity detection. The release criteria were: RCP ≥98%, pH 4.5–5.5, endotoxin ≤5 EU/mL (Limulus Amebocyte Lysate test), and a clear, particulate-free solution. *In vitro* stability was evaluated by incubating the product in phosphate-buffered saline (PBS) and mouse serum at 37 °C, with RCP measured at 1, 24, 72, and 168 h; RCP ≥90% was considered stable (Hooijman et al., 2024).

### 2.3 Animal model and study groups

#### 2.3.1 Animals and tumor inoculation

Male BALB/c nude mice (6–8 weeks old) were housed under standard specific pathogen-free conditions. PSMA-positive C4-2 human prostate cancer cells were cultured in RPMI-1640 medium supplemented with 10% fetal bovine serum. For xenograft establishment, 5 × 10^6^ cells in a 1:1 mixture of serum-free medium and Matrigel were injected subcutaneously into the right axilla (Luo et al., 2021). Tumor volume (V) was calculated as V = (length × width^2^)/2. Animals with tumors reaching 100–150 mm^3^ were enrolled in the study. PSMA expression was confirmed in an initial cohort by immunohistochemistry.

#### 2.3.2 Group allocation and dosing

Animals were randomized using a block design (block size = 4), stratified by baseline tumor volume. The study consisted of two cohorts:

Dosimetry Group (n = 32): Received a single intravenous (i.v.) injection of 5 kBq ^225^Ac-PSMA-617 (0.5 nmol peptide). Four animals were euthanized at each of eight time points (0.5, 2, 6, 24, 48, 96, 168, 336 h) for biodistribution.

Therapy Group (n = 24): Randomized into two arms (n = 12/arm). The treatment arm received a single i.v. injection of 40 kBq ^225^Ac-PSMA-617 (0.5 nmol peptide). The control arm received a volume-matched vehicle solution.

The therapeutic activity of 40 kBq per mouse was selected based on prior published studies evaluating ^225^Ac-PSMA-617 in similar xenograft models, which demonstrated antitumor efficacy with acceptable tolerability in this activity range (Juzeniene et al., 2021; Ma et al., 2022). This dose also represents an approximately 8-fold escalation above the tracer dose (5 kBq) used for dosimetry, allowing proportional scaling of absorbed dose estimates while maintaining the same peptide mass (0.5 nmol) across both groups to minimize receptor occupancy effects.

For all injections, syringe activity was measured pre- and post-injection via a dose calibrator. The actual injected activity was calculated by difference, with a residual activity threshold of ≤5% (Zelechowska-Matysiak et al., 2023). Blinding was maintained for tumor measurement and pathological analysis.

### 2.4 Biodistribution and dosimetry

#### 2.4.1 Sample collection and radioactivity measurement

At each dosimetry time point, animals were anesthetized, and systemic perfusion with saline was performed via cardiac puncture. Tumors, kidneys, salivary glands (parotid, submandibular, sublingual), and other major organs were harvested, weighed, and collected in pre-weighed tubes. To allow the ^225^Ac decay chain to reach approximate transient equilibrium for reliable gamma counting, samples were stored at room temperature for 24 h before measurement (Kruijff et al., 2019). Radioactivity was measured using a calibrated gamma counter with energy windows set for the 218 keV (^221^Fr) and 440 keV (^213^Bi) peaks. Counts were decay-corrected to the time of injection using the ^225^Ac half-life of 9.92 days (National Nuclear Data Center, 2019) and normalized to percentage of injected activity per gram (%IA/g) and per organ (%IA/organ).

#### 2.4.2 Dosimetric calculations

Time-activity curves were generated for the tumor, kidneys (combined), and each salivary gland. Data were fitted with a bi-exponential function, A(t) = A_1_e^(-k_1_t)^ + A_2_e^(-k_2_t)^, using weighted least-squares regression (Ivashchenko et al., 2024). The time-integrated activity (Ã, MBq·h) was calculated by integrating the fitted function (Siegel et al., 1999).

The absorbed dose D (Gy) to each target tissue was calculated using the MIRD formalism: D = (Ã · Σ Δᵢ · φᵢ)/m, where Σ Δᵢ · φᵢ represents the total energy deposited per decay. For the short-range alpha and beta particles in mouse-sized tissues, the absorbed fraction (φ) was assumed to be 1. The mean energy emitted per nuclear transition for the complete ^225^Ac decay chain (^225^Ac→^221^Fr→^217^At→^213^Bi→^213^Po→^209^Pb→^209^Bi) was taken from established nuclear data tables (Jalloul et al., 2023). Individual organ masses were used for dose calculations.

#### 2.4.3 Uncertainty and sensitivity analysis

Uncertainty in absorbed dose, originating from counting statistics, fitting parameters, and mass measurement, was propagated using a parametric bootstrap method (1,000 iterations). The sensitivity of dose estimates to uncertainties in the nuclear decay data (E_total) was assessed by a ±5% perturbation (Bé et al., 2015).

### 2.5 Efficacy and toxicity assessments

#### 2.5.1 Antitumor efficacy

In the therapy group, tumor volume was measured thrice weekly for 28 days. Efficacy was evaluated using two primary endpoints: (1) Tumor Growth Inhibition (TGI) on Day 28, calculated as;

$$\text{TGI}=1‐\frac{\text{AU}C_{\text{Treatment}}}{\text{AU}C_{\text{Control}}}$$

where AUC is the area under the curve of log-transformed tumor volume over time (Wu and Houghton, 2010); and (2) Tumor Growth Delay, defined as the time for an individual tumor to reach 500 mm^3^, analyzed by Kaplan-Meier survival curves and the log-rank test.

#### 2.5.2 Renal toxicity

Renal function was assessed by measuring blood urea nitrogen (BUN) and serum creatinine at baseline and on Days 14, 28, and 56. On Day 56, kidneys were harvested for histopathology. Paraffin-embedded sections were stained with hematoxylin and eosin (H&E) and periodic acid–Schiff (PAS). Tubular injury in the renal cortex was scored semi-quantitatively (0–4) by two blinded pathologists, using established criteria (van Timmeren et al., 2006).

#### 2.5.3 Salivary gland toxicity

Salivary gland function was assessed via a pilocarpine-stimulated secretion test at baseline and on Days 7, 14, and 28. Saliva was collected for 10 min after an intraperitoneal injection of pilocarpine (1 mg/kg), and the flow rate was normalized to body weight (µL/min/g). On Day 28, salivary glands were collected for H&E staining and scored (0–4) for acinar atrophy, ductal dilation, and inflammation.

### 2.6 Therapeutic index and integrated analysis

The therapeutic index (TI) for each organ at risk (OAR) was defined as the ratio of tumor absorbed dose to OAR absorbed dose:

$$TI_{\text{tumor}/\text{kidney}}=\frac{D_{\text{tumor}}}{D_{\text{kidney}}},TI_{\text{tumor}/\text{salivary}}=\frac{D_{\text{tumor}}}{D_{\text{salivary}}}$$

where *D* represents the absorbed dose (Gy), the overall TI for an animal was conservatively defined as the minimum of these values:

$$TI_{\min}=\left(TI_{\text{tumor}/\text{kidney}},TI_{\text{tumor}/\text{salivary}}\right)$$

A ${\text{TI}}_{\min}>1$ indicates a positive therapeutic window (Sgouros et al., 2010).

To establish dose-response relationships, Spearman rank correlation analysis was performed within the treatment group between: (i) tumor dose and Day 28 TGI, (ii) kidney dose and Day 56 renal histopathology score, and (iii) salivary gland dose and the maximum percentage decrease in stimulated salivary flow.

### 2.7 Statistical analysis

All analyses were performed using R (v4.3.0) and GraphPad Prism (v10.0). Data are presented as mean ± standard deviation or geometric mean with 95% confidence interval, as appropriate. Group comparisons for TGI and biochemical markers over time used mixed-effects models. The log-rank test was used for survival analysis. Correlation strength was reported using Spearman’s ρ. The Holm-Bonferroni method was applied to correct for multiple comparisons. A two-sided p-value <0.05 was considered statistically significant. To assess the translational relevance of our preclinical findings, tumor-to-kidney dose ratios were compared with published clinical dosimetry data for ^225^Ac-PSMA-617 (Kratochwil et al., 2016; Kratochwil et al., 2018; Sathekge et al., 2019).

### 2.8 Ethical statement

All animal procedures were conducted in accordance with institutional guidelines and were approved by the Institutional Animal Care and Use Committee (IACUC) of Macau University of Science and Technology, Macau SAR, China (Approval No: 2025-S-0033).

## 3 Results

### 3.1 Radiopharmaceutical synthesis and quality control

**FIGURE 1:**
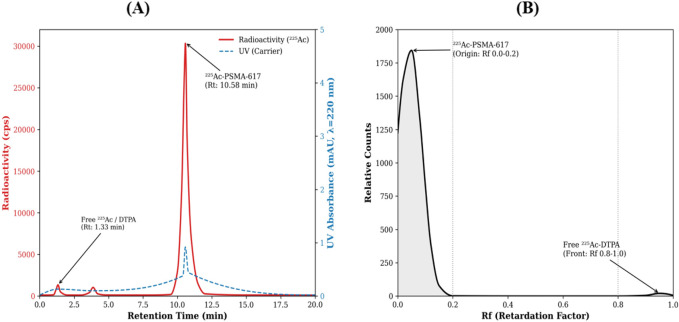
Representative quality control chromatograms of ^225^Ac-PSMA-617. **(A)** Reverse-phase HPLC radiochromatogram (UV trace at 220 nm in grey, radioactivity trace in red). The central peak corresponds to ^225^Ac-PSMA-617. **(B)** Radio-thin-layer chromatography (iTLC-SG) scan showing the product at the origin.

All eight synthesized batches of ^225^Ac-PSMA-617 met the predefined release criteria. Representative chromatograms confirmed a single dominant radioactive species corresponding to ^225^Ac-PSMA-617 (HPLC retention time: 10.58 min; iTLC Rf: 0.0–0.2), with minimal free ^225^Ac (<1%) (Figures 1A,B). The product demonstrated high *in vitro* stability in both PBS and mouse serum at 37 °C, maintaining RCP >90% through the 168-h assessment period. A summary of quality control and stability results for all batches is provided in Supplementary Table S2.

### 3.2 Dosing accuracy and model compliance

The accuracy of intravenous administration was validated for all groups (Table 1). The actual injected activities for the dosimetry (5 kBq planned) and treatment (40 kBq planned) groups closely matched the target values, with mean residual activity in the syringes consistently below the 5% acceptance threshold.

**TABLE 1:** Verification of injected activity and dosing compliance.

Group	Planned injected activity (kBq)	Actual injected activity (kBq, mean ± SD)	Residual ratio (%, mean ± SD)	Injection volume (μL, mean ± SD)
Dosimetry group (n = 32)	5	4.92 ± 0.19	2.31 ± 0.88	100.74 ± 1.91
Treatment group (n = 12)	40	39.46 ± 1.28	2.74 ± 1.04	100.92 ± 1.83
Control group (n = 12)	0	0.004 ± 0.003	—	100.61 ± 2.07

Residual ratio threshold ≤5% was considered acceptable.

### 3.3 Biodistribution and time-activity curves

**FIGURE 2:**
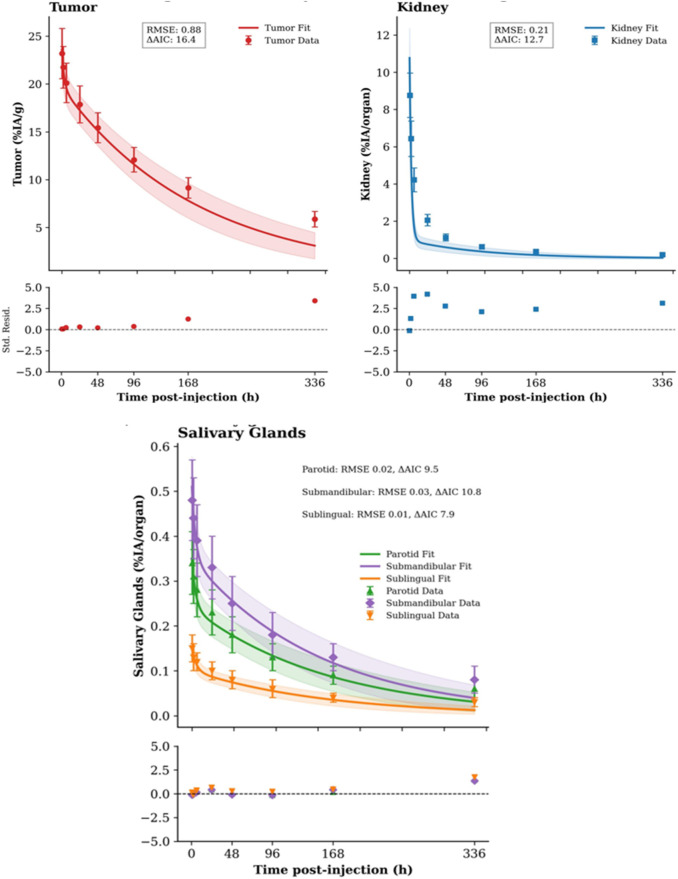
Pharmacokinetics of ^225^Ac-PSMA-617. Time-activity curves (mean ± SD, n = 4 per time point) for tumor, kidneys, and parotid gland after a 5 kBq injection. Solid lines represent the bi-exponential model fit. Insets show residual plots for each fit.

Biodistribution data from the dosimetry group revealed distinct pharmacokinetic profiles (Figure 2). Tumor uptake was high and persistent, while renal uptake peaked early and cleared rapidly. Salivary glands (parotid, submandibular, sublingual) exhibited lower but prolonged retention. Time-activity curves were best described by a bi-exponential model, which provided a superior fit over a mono-exponential model (ΔAIC range: 7.9–16.4).

### 3.4 Dosimetry, uncertainty, and therapeutic index

Dosimetric calculations based on the bi-exponential fits yielded high absorbed dose coefficients per unit administered activity, reflecting the potent energy deposition of the ^225^Ac decay chain (Table 2). The tumor exhibited the highest dose coefficient. Notably, the parotid gland received a higher mean dose coefficient than the kidneys.

**TABLE 2:** Dosimetry of ^225^Ac-PSMA-617: Time-integrated activity and absorbed dose coefficients.

Tissue/Organ	Time-integrated activity coefficient (MBq·h per MBq administered)	Absorbed dose coefficient (Gy per MBq administered)
Tumor	4.00 (3.14–5.00)	588 (462–736)
Kidneys (both)	1.20 (1.00–1.40)	58 (48–76)
Parotid gland	0.40 (0.31–0.49)	112 (88–138)
Submandibular gland	0.60 (0.47–0.73)	46 (36–56)
Sublingual gland	0.20 (0.15–0.25)	68 (52–84)

The high Gy/MBq, coefficients reflect the substantial energy deposition from the complete α-decay chain of ^225^Ac. for reference, a 5 kBq (0.005 MBq) injection delivers the following mean absorbed doses: Tumor = 2.94 Gy, Kidneys = 0.29 Gy, Parotid = 0.56 Gy—coefficients derived from a 5 kBq administration. Values in parentheses represent 95% confidence intervals.

**FIGURE 3:**
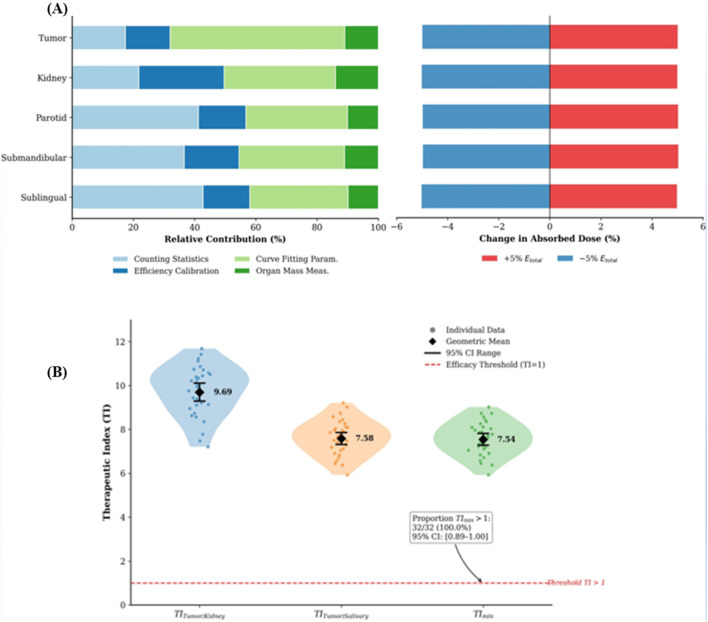
Dosimetric analysis of ^225^Ac-PSMA-617. **(A)** Analysis of absorbed dose uncertainty and sensitivity. Left: Variance decomposition showing the contribution of different error sources to the total uncertainty for each tissue (n = 1,000 bootstrap iterations). Right: Sensitivity of the mean dose to a ±5% change in total decay energy (E_total). **(B)** Distribution of the therapeutic index (TI) for individual animals (n = 32). TI for kidneys (D_tumor/D_kidney) and salivary glands (D_tumor/D_salivary) are shown, where D_salivary is the mean dose across parotid, submandibular, and sublingual glands. TI_min = min (TI_tumor/kidney, TI_tumor/salivary). Dashed line at TI = 1.

Uncertainty analysis indicated that dose estimates for tumors and kidneys were most sensitive to curve-fitting parameters, while salivary gland doses were more influenced by counting statistics (Figure 3A). The therapeutic index (TI), calculated as the ratio of tumor to organ-at-risk dose, was favorable. Both the TI for kidneys (TI_tumor/kidney) and for salivary glands (TI_tumor/salivary) were substantially greater than 1 for all animals, confirming a consistent therapeutic window (Figure 3B).

### 3.5 Antitumor efficacy of ^225^Ac-PSMA-617

**FIGURE 4:**
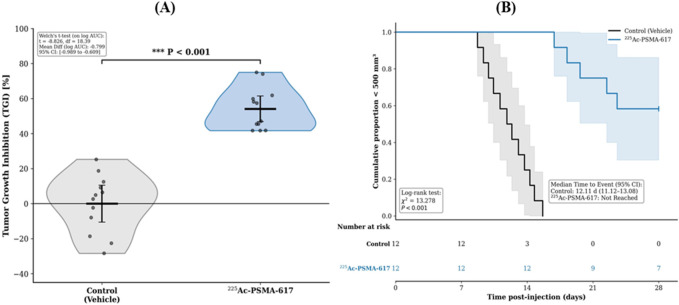
Antitumor efficacy of ^225^Ac-PSMA-617. **(A)** Tumor Growth Inhibition (TGI) on Day 28 in mice treated with a single 40 kBq dose versus vehicle control. Data represent mean ± SD. ***P < 0.001 by Welch’s t-test. **(B)** Kaplan-Meier curves showing tumor growth delay, defined as the time to reach 500 mm^3^. ***P < 0.001 by log-rank test.

Treatment with a single 40 kBq dose of ^225^Ac-PSMA-617 resulted in significant tumor control. The tumor growth inhibition (TGI) on Day 28 was significantly greater in the treatment group compared to the vehicle control group (P < 0.001, Welch’s t-test) (Figure 4A). This was corroborated by a marked delay in tumor progression, with the time to reach a volume of 500 mm^3^ being significantly longer in treated animals (log-rank P < 0.001) (Figure 4B).

### 3.6 Organ-specific toxicity and systemic tolerability

#### 3.6.1 Renal and salivary gland toxicity

**FIGURE 5:**
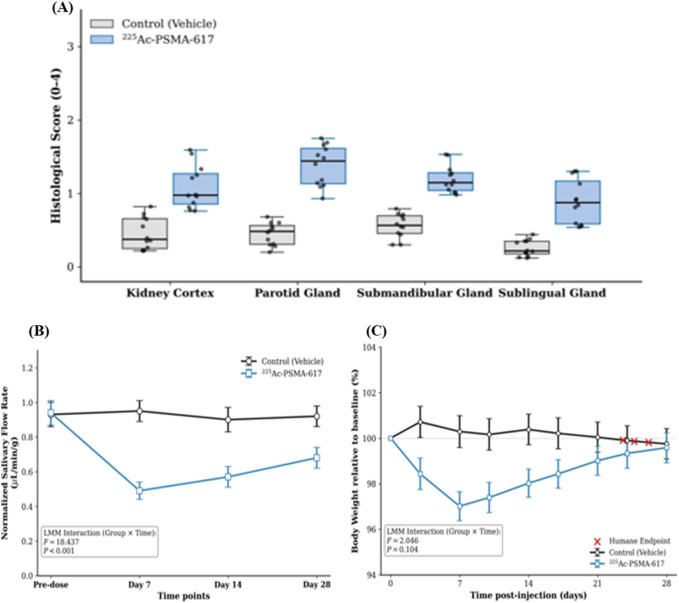
Assessment of organ-specific toxicity and systemic tolerability following ^225^Ac-PSMA-617 therapy. **(A)** Semi-quantitative histopathology scores for kidney (assessed at Day 56) and salivary glands (parotid, submandibular, sublingual; assessed at Day 28). Each point represents one animal; horizontal bars indicate the group median. **P < 0.01, treatment vs. control (Mann-Whitney U test). **(B)** Time course of pilocarpine-stimulated salivary flow rate, normalized to body weight. Data are presented as mean ± standard error of the mean (SEM). ***P < 0.001 for group × time interaction (linear mixed-effects model). **(C)** Mean body weight change (percentage relative to baseline) over time in the treatment and vehicle control groups. Error bars represent SEM. No significant difference was observed between groups (P = 0.104 for the group × time interaction in the linear mixed-effects model).

No significant changes in renal function, as measured by blood urea nitrogen (BUN) and serum creatinine, were observed in the treatment group compared to controls at any time point up to Day 56 (all P > 0.05) (Table 3). However, histopathological analysis at Day 56 revealed mild-to-moderate tubular injury in the renal cortex of treated animals, with significantly higher scores than in controls (mean ± SD: 1.06 ± 0.26 vs. 0.48 ± 0.22; median: 1.02 vs. 0.45; P < 0.01) (Figure 5A).

**TABLE 3:** Renal function biochemical indices over time.

Time point	Blood urea nitrogen (mmol/L)			Serum creatinine (μmol/L)		
	Control group	Treatment group	Difference (95% CI)	Control group	Treatment group	Difference (95% CI)
Baseline	7.42 ± 0.89	7.39 ± 0.92	−0.03 (−0.71–0.65)	36.71 ± 4.93	36.54 ± 5.28	−0.17 (−3.42–3.08)
Day 14	7.58 ± 0.94	7.62 ± 0.88	0.04 (−0.66–0.73)	37.09 ± 5.02	38.12 ± 5.26	1.03 (−2.21–4.31)
Day 28	7.67 ± 0.91	7.81 ± 0.95	0.14 (−0.59–0.86)	37.88 ± 5.36	39.42 ± 5.51	1.54 (−1.87–4.89)
Day 56	7.51 ± 0.97	7.92 ± 1.02	0.41 (0.35–1.16)	38.01 ± 5.44	40.28 ± 5.92	2.27 (−1.32–5.74)

BUN, Group effect: P = 0.145; Time effect: P = 0.262; Interaction: P = 0.476. Creatinine – Group effect: P = 0.101; Time effect: P = 0.139; Interaction: P = 0.409. Data are mean ± SD., Statistical analysis used a linear mixed-effects model.

In contrast, salivary gland function was acutely and persistently impaired. Pilocarpine-stimulated salivary flow, normalized to body weight, showed a significant and sustained decrease in the treatment group starting at Day 7, with no recovery to baseline by Day 28 (group × time interaction, P < 0.001) (Figure 5B). Histological assessment at Day 28 confirmed mild-to-moderate salivary gland injury, with higher scores in treated animals than in controls across all three glands: parotid (1.31 ± 0.29 vs. 0.47 ± 0.17), submandibular (1.19 ± 0.19 vs. 0.57 ± 0.17), and sublingual (0.93 ± 0.33 vs. 0.27 ± 0.12), consistent with the observed functional deficit (Figure 5A).

#### 3.6.2 Systemic tolerability

Body weight, a marker of systemic toxicity, remained stable in both groups throughout the study, with no significant difference in weight change over time (P = 0.104) (Figure 5C), indicating good overall tolerability of the 40 kBq regimen.

### 3.7 Dose-response correlation analysis

**FIGURE 6:**
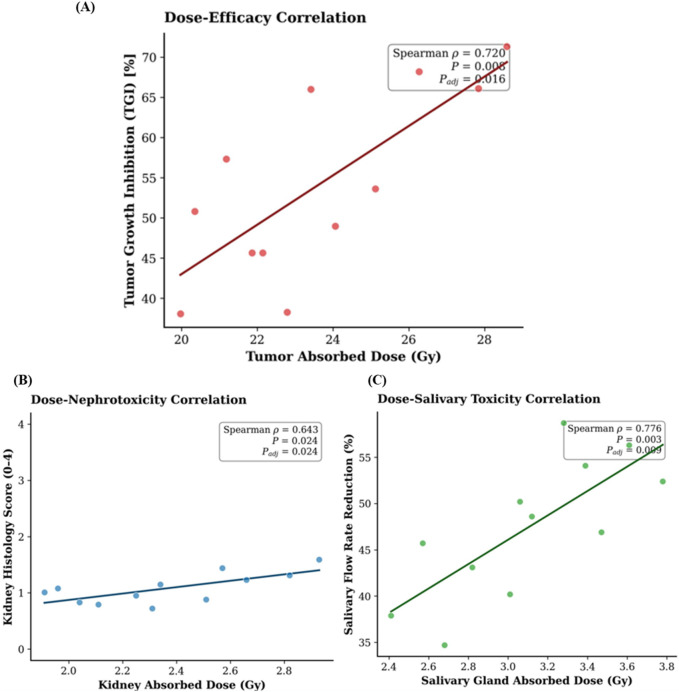
Dose-response correlations. Scatter plots showing the relationship between absorbed dose and biological endpoints: **(A)** tumor dose vs. tumor growth inhibition (TGI), **(B)** kidney dose vs. renal histopathology score, and **(C)** salivary gland dose (mean of three glands) vs. percentage decrease in salivary flow. Absorbed doses were calculated per the MIRD formalism. Spearman’s ρ and adjusted P-values (Holm-Bonferroni correction) are shown. Solid lines indicate linear regression fits for visualization.

Significant positive correlations were found between absorbed dose and key biological endpoints (Figure 6). The tumor dose correlated strongly with therapeutic efficacy (TGI; ρ = 0.720, P_adj <0.05) (Figure 6A), the kidney dose with renal histopathology score (ρ = 0.643, P_adj <0.05) (Figure 6B), and the salivary gland dose with the percentage decrease in salivary flow (ρ = 0.776, P_adj <0.05) (Figure 6C). A sub-analysis using parotid gland dose alone yielded a similarly strong correlation with the percentage decrease in salivary flow (ρ = 0.751, P_adj <0.001), confirming that the parotid gland is a primary driver of the observed functional impairment. These correlations provide a quantitative link between the physical dosimetry and the observed efficacy and toxicity profiles.

## 4 Discussion

This preclinical study provides a comprehensive, quantitative evaluation of the dosimetry and therapeutic window of ^225^Ac-PSMA-617, integrating detailed organ-level absorbed dose calculations with longitudinal assessments of antitumor efficacy and organ-specific toxicity. The principal findings demonstrate that a single administration achieves a high tumor absorbed dose and a favorable therapeutic index (TI > 1), resulting in significant tumor growth inhibition with acceptable systemic tolerability. However, an apparent dissociation between organs at risk was observed: while renal function remained stable, the salivary glands emerged as the primary site of dose-limiting toxicity, exhibiting a profound and sustained functional deficit that correlated strongly with the absorbed dose.

The high radiochemical purity and stability of our ^225^Ac-PSMA-617 batches ensured that the observed biodistribution and toxicity profiles were attributable to the targeted radiopharmaceutical rather than free radiometal, a critical prerequisite for interpreting alpha-emitter dosimetry (Hooijman et al., 2024; Thakral et al., 2021). The distinct pharmacokinetic profiles, persistent tumor retention, rapid renal clearance, and prolonged salivary gland retention directly informed the absorbed dose calculations. Notably, the parotid gland received a higher dose coefficient than the kidneys, a finding that underscores the importance of gland-specific dosimetry even when macroscopic uptake appears low (Tönnesmann et al., 2019). Our use of a bi-exponential model and a rigorous uncertainty framework, including sensitivity analysis for nuclear decay data, strengthens the reliability of these dose estimates and facilitates future cross-study comparisons (Ivashchenko et al., 2024; Bé et al., 2015).

The robust antitumor efficacy observed at the 40 kBq dose level aligns with the potent cytotoxicity expected from alpha-particle irradiation. It validates the high tumor dose coefficients calculated from our dosimetry (Juzeniene et al., 2021). More importantly, the integration of dosimetry with efficacy and toxicity endpoints yielded significant dose-response correlations. The strong correlation between tumor dose and growth inhibition (ρ = 0.720) provides a quantitative scaling factor for efficacy, while the correlations for kidney (ρ = 0.643) and salivary gland (ρ = 0.776) doses with their respective toxicity markers establish a predictive framework for organ risk. These correlations move beyond simple safety reporting and provide a mechanistic link between physical dose deposition and biological effect, which is essential for rational dose optimization (O’Donoghue et al., 2022).

The differential toxicity profile between kidneys and salivary glands offers critical insights. The absence of significant renal impairment despite measurable histopathological changes is consistent with the known limitations of blood urea nitrogen and creatinine in detecting early tubular injury (Li et al., 2024). This “structural injury before functional decline” pattern suggests that conventional renal biomarkers may be insufficient for monitoring alpha-emitter therapy, warranting the investigation of more sensitive urinary biomarkers, such as KIM-1 or NGAL, in future studies (van Timmeren et al., 2006). In stark contrast, salivary gland function served as a highly sensitive and early indicator of radiation injury. The profound and persistent reduction in stimulated salivary flow, strongly correlated with dose, highlights the unique vulnerability of salivary acinar cells to the dense ionization tracks of alpha particles (Muniz et al., 2024). This finding positions salivary gland function, rather than histology alone, as a clinically translatable functional endpoint for defining dose-limiting toxicity in ^225^Ac-PSMA-617 therapy. Notably, a sub-analysis using parotid gland dose alone confirmed a similarly strong correlation with functional impairment (ρ = 0.751), indicating that the parotid gland is a primary contributor to the observed xerostomia and supporting the use of mean salivary gland dose as a robust predictor of overall salivary toxicity.

The therapeutic index (TI), particularly the TI_min metric, successfully integrated these complex trade-offs into a single, interpretable value (Sgouros et al., 2010). A TI_min >1 across all animals confirms the existence of a therapeutic window under these experimental conditions. However, the fact that the salivary gland dose is more frequently limited than the renal dose clearly identifies xerostomia as the primary clinical challenge for activity escalation. This evidence-based identification of the dose-limiting organ is a key outcome of our integrated approach. It directly informs clinical trial design, suggesting that salivary gland-protective strategies (e.g., cooling, gland massage, or receptor-blocking agents) may be more effective than generalized dose reduction in improving the therapeutic window (Heynickx et al., 2023).

The therapeutic index (TI), particularly the TI_min metric, successfully integrated these complex trade-offs into a single, interpretable value (Sgouros et al., 2010). A TI_min >1 across all animals confirms the existence of a therapeutic window under these experimental conditions. To contextualize our preclinical findings, we compared the tumor-to-kidney absorbed dose ratio observed in our study with available clinical dosimetry data for ^225^Ac-PSMA-617. In the present study, the tumor-to-kidney dose ratio was approximately 10:1, calculated from the absorbed dose coefficients of 588 Gy/GBq for tumor and 58 Gy/GBq for kidneys. This ratio is somewhat higher than those reported in clinical studies, where tumor-to-kidney ratios have typically ranged from 4:1 to 8:1 (Kratochwil et al., 2016; Kratochwil et al., 2018; Sathekge et al., 2019; Ma et al., 2022). The more favorable ratio in our preclinical model may reflect several factors, including the relatively small and homogeneous tumor xenografts, the absence of prior therapies that could alter renal physiology, and species-specific differences in PSMA expression and renal handling of radioligands. Nevertheless, the consistent observation across both preclinical and clinical settings that tumor dose substantially exceeds renal dose supports the existence of a therapeutic window for ^225^Ac-PSMA-617. These comparisons underscore the value of preclinical dosimetry for predicting clinical performance while highlighting the need for cautious translation of absolute dose values. However, the fact that the salivary gland dose is more frequently limited than the renal dose clearly identifies xerostomia as the primary clinical challenge for activity escalation. This evidence-based identification of the dose-limiting organ is a key outcome of our integrated approach. It directly informs clinical trial design, suggesting that salivary gland-protective strategies (e.g., cooling, gland massage, or receptor-blocking agents) may be more effective than generalized dose reduction in improving the therapeutic window (Heynickx et al., 2023).

### 4.1 Limitations and future directions

Our study has several limitations that point to valuable future research. First, dosimetry was based on a macroscopic MIRD model with simplified assumptions (e.g., a homogeneous activity distribution and an absorbed fraction of 1 for alpha particles). This approach does not capture microdosimetric heterogeneity, particularly relevant for small structures such as salivary gland ducts and acini, where local “hot spots” from decaying atoms may drive toxicity beyond predictions based on the mean absorbed dose (Tronchin et al., 2022). Furthermore, our model does not account for potential daughter nuclide redistribution following alpha recoil. Each decay in the ^225^Ac cascade imparts significant recoil energy (∼100 keV) to the daughter nucleus, which can disrupt chelation and allow redistribution of daughters such as ^221^Fr or ^213^Bi away from the original binding site (Kruijff et al., 2019). This effect may disproportionately impact the salivary glands due to their small size and complex glandular architecture, where localized “hot spots” from redistributed daughters could amplify functional injury beyond that predicted by the mean organ dose. In contrast, the larger and more homogeneous parenchyma of the kidneys may be less susceptible to such microdosimetric extremes, potentially explaining the discordance between preserved renal function and mild histological changes observed in our study. Quantifying these effects would require advanced alpha camera imaging or cellular-level Monte Carlo simulations beyond the present scope.

Second, the single activity level (40 kBq) for therapy limits our understanding of the complete dose-response curve. Future studies employing multiple activity levels or fractionated regimens are needed to establish potential toxicity thresholds and efficacy saturation points (Zanzonico, 2025). Third, while our 56-day observation captured subclinical renal damage, the long-term progression of alpha-particle-induced nephropathy remains undefined. Alpha-induced renal injury can be latent and progressive, with potential for delayed functional decline beyond the observation window of this study (Li et al., 2024). The mild-to-moderate tubular injury observed at Day 56 may represent either a stable, subclinical finding or an early stage of progressive nephropathy that could manifest functionally at later time points. Extended follow-up studies with serial histology and sensitive urinary biomarkers (e.g., NGAL, KIM-1) are necessary to characterize fully the temporal evolution of renal injury and determine whether the structural changes observed represent a precursor to progressive nephropathy. Finally, using a single PSMA-high xenograft model and a single dosing schedule limits generalizability. Evaluating the therapeutic index in models with heterogeneous or lower PSMA expression and exploring optimized fractionation schedules will be crucial for understanding the clinical applicability of these findings across diverse patient populations (Sathekge et al., 2021).

## 5 Conclusion

In conclusion, this integrated dosimetry-efficacy-toxicity study in a preclinical model demonstrates that ^225^Ac-PSMA-617 delivers a therapeutically effective tumor dose with a quantifiable safety margin (TI > 1). The salivary glands, not the kidneys, were identified as the primary dose-limiting organ based on a profound and dose-correlated functional deficit. The established correlations between absorbed dose and biological endpoints provide a quantitative foundation for personalized dose planning. To advance this therapy, future efforts should focus on validating these dose-response relationships in multi-dose studies, developing strategies to mitigate salivary gland toxicity, and employing more sensitive biomarkers to monitor subclinical organ injury.

## Data Availability

The original contributions presented in the study are included in the article/Supplementary Material, further inquiries can be directed to the corresponding author.
