# Supplementary material for: Salivary glands as the primary dose-limiting organ: an integrated dosimetry, efficacy, and toxicity study of 225Ac-PSMA-617 in prostate cancer xenografts
Source: Front Pharmacol. 2026 Apr 10;17:1807913. doi: 10.3389/fphar.2026.1807913 (PMC13106407; doi:10.3389/fphar.2026.1807913)
Supplement: Supplementary file 1 [file Table1.pdf]

**Table S1.** List of materials, reagents, cell lines, animals, and major equipment used in the study, including suppliers and catalog numbers.

| Name of Material/Equipment | Company | Catalog Number | Comments/Description |
|---|---|---|---|
| Actinium-225 (as nitrate solution, typically supplied in dilute HCl) | Eckert & Ziegler Medical | N/A | $^{225}Ac$ supply for radiolabeling; ordering identifiers vary by contract and regulatory approvals. |
| PSMA-617 (DOTA-PSMA-617) | MedChemExpress (MCE) | HY-117410 | PSMA-617 (DOTA chelator) used for $^{225}Ac$ labeling. |
| Diethylenetriaminepentaacetic acid (DTPA) | Sigma-Aldrich (Merck) | D6518 | Used to chelate unbound $^{225}Ac$ after labeling (DTPA "chase") |
| iTLC-SG chromatography strips | Agilent Technologies | SGI0001 | iTLC-SG strips for radio-TLC QC. |
| Radio-TLC scanner | Company | Catalog Number | Comments/Description |
| Radio-TLC scanner | Eckert & Ziegler (EZAG) | AR-2000 | AR-2000 radio-TLC imaging/scanning system for iTLC readout. |
| HPLC column, C18, 250 × 4.6 mm, 5 µm | Agilent Technologies | 959990-902 | ZORBAX Eclipse Plus C18 column matching the stated dimensions. |
| Trifluoroacetic acid (TFA), HPLC grade | Sigma-Aldrich (Merck) | 302031 | Mobile phase additive. |
| Acetonitrile, gradient/HPLC grade | Sigma-Aldrich (Merck) | 34851 | Mobile phase organic solvent for RP-HPLC gradients. |
| Sterile syringe filter, 0.22 µm (PVDF) | MilliporeSigma (Merck Millipore) | SLGV033RS | 0.22 µm sterilizing filtration of final injectable formulation. |
| Matrigel® Basement Membrane Matrix (LDEV-Free) | Corning | 356234 | Mixed 1:1 with cells for subcutaneous xenograft implantation. |
| Pilocarpine hydrochloride | Sigma-Aldrich (Merck) | P6503 | Used for pilocarpine-induced salivation testing in mice. |
| BALB/c nude mice (male, 6–8 weeks) | Beijing Vital River Laboratory Animal Technology Co., Ltd. | Strain code 401 | BALB/c Nude mice for PSMA-positive prostate cancer xenografts. |
| C4-2 human prostate cancer cell line | ATCC | CRL-3314 | C4-2 cell line (base line; PSMA-positive in many settings—verify if additional stable PSMA engineering was performed). |
| RPMI-1640 medium | Gibco (Thermo Fisher Scientific) | 11875-093 | Cell culture medium used for C4-2 culture in the described protocol. |
| Fetal Bovine Serum (FBS) | Gibco (Thermo Fisher Scientific) | 10099-141 | Used at 10% (v/v) for cell culture. |
| Penicillin–Streptomycin (10,000 U/mL) | Gibco (Thermo Fisher Scientific) | 15140-122 | Used at 1% (v/v) for routine cell culture. |

| | | | |
|---|---|---|---|
| Gel-clot endotoxin test kit (TAL/LAL) | Xiamen Bioendo Technology Co., Ltd. | G170030 | Example Bioendo gel-clot endotoxin kit catalog ID. |
| Dose calibrator (activity meter) | Capintec (Mirion) | CRC-55tR (model) | Common nuclear medicine dose calibrator for syringe activity verification. |
| Automatic gamma counter (NaI(Tl)) | Revvity (formerly PerkinElmer) | Wizard2 2480 (model) | NaI(Tl) gamma counter commonly used for sample counting. |
| HPGe gamma spectrometer (HPGe detector) | Mirion (Canberra) | SEGe series | HPGe detector family; datasheet lists GC-model options. |
| Radio-HPLC detector (online radiation detector) | Eckert & Ziegler / BioScan | FlowCount PRO (model) | Online radioactivity detector for HPLC . |
| Analytical balance (0.1 mg readability) | METTLER TOLEDO | ME204 (model) | Analytical balance suitable for organ/sample weighing. |
| Digital caliper (0–150 mm) | Mitutoyo | 500-196-30 | Digital caliper commonly used for tumor size measurements. |
| $CO_2$ euthanasia chamber/system | Yuyan Instrument Co., Ltd. | CL-1000L | $CO_2$ euthanasia chamber model. |

**Table S2.** Radiochemical purity and stability profile of synthesized $^{225}$Ac-PSMA-617 batches.

| Parameter / Batch | A01 | A02 | A03 | A04 | A05 | A06 | A07 | A08 |
|---|---|---|---|---|---|---|---|---|
| **Synthesis & Release** | | | | | | | | |
| Loaded activity (MBq) | 3.92 | 4.35 | 5.14 | 3.47 | 4.88 | 2.96 | 5.63 | 4.21 |
| Uncorrected yield (%) | 82.73 | 78.91 | 85.63 | 74.82 | 83.41 | 80.34 | 86.12 | 79.54 |
| RCP at release (%) | 99.14 | 98.47 | 99.36 | 98.05 | 99.08 | 98.93 | 99.41 | 98.62 |
| pH | 5.08 | 4.92 | 5.11 | 4.78 | 5.06 | 4.85 | 5.19 | 4.96 |
| Endotoxin (EU/mL) | 0.08 | 0.14 | 0.008 | 0.22 | 0.18 | 0.009 | 0.27 | 0.12 |
| **Stability in PBS, 37°C** | | | | | | | | |
| RCP at 1 h (%) | 98.97 | 99.02 | 99.28 | 98.73 | 99.01 | 98.88 | 99.33 | 98.95 |
| RCP at 24 h (%) | 97.58 | 97.21 | 97.83 | 97.05 | 97.69 | 97.23 | 97.98 | 97.37 |
| RCP at 72 h (%) | 96.41 | 95.99 | 96.72 | 95.79 | 96.36 | 95.86 | 96.64 | 96.02 |
| RCP at 168 h (%) | 95.12 | 94.67 | 95.34 | 94.88 | 95.07 | 94.74 | 95.45 | 94.93 |
| RCP at 1 h (%) | 98.97 | 99.02 | 99.28 | 98.73 | 99.01 | 98.88 | 99.33 | 98.95 |
| **Stability in Serum, 37°C** | | | | | | | | |
| RCP at 1 h (%) | 98.71 | 98.33 | 98.86 | 98.19 | 98.64 | 98.27 | 98.92 | 98.42 |
| RCP at 24 h (%) | 96.05 | 95.68 | 96.18 | 95.44 | 96.11 | 95.79 | 96.23 | 95.94 |
| RCP at 72 h (%) | 94.18 | 93.24 | 94.09 | 93.16 | 93.97 | 93.42 | 94.13 | 93.59 |
| RCP at 168 h (%) | 91.84 | 91.47 | 92.31 | 91.62 | 92.16 | 91.93 | 92.39 | 92.08 |

**Note:** RCP = radiochemical purity; EU = endotoxin units. Release criteria: RCP ≥ 98%, pH 4.5–5.5, endotoxin ≤ 5 EU/mL. Stability was assessed in PBS and mouse serum at 37°C; RCP ≥ 90% was considered stable.
